# Mapping Five Years of #FOAMed: Trends, Engagement, and Shifting Topics on Twitter/X

**DOI:** 10.5811/westjem.47392

**Published:** 2025-12-19

**Authors:** Ertuğ Günsoy, Ahmet Aykut, Cem Yildirim, Mehmet Veysel Öncül

**Affiliations:** University of Health Sciences, Van Education and Research Hospital, Department of Emergency Medicine, Van, Türkiye

## Abstract

**Introduction:**

Free Open Access Medical Education (FOAMed) has emerged as a prominent component of online medical communication, with X (formerly Twitter) serving as an active hub for professional exchange among clinicians. Despite its reach and influence, few longitudinal studies have examined how FOAMed content and engagement patterns evolve over time. In this study we aimed to analyze thematic shifts and user interaction trends in #FOAMed tweets over a five-year period.

**Methods:**

We conducted a retrospective bibliometric and natural language processing (NLP) study of 6,000 high-engagement, English-language tweets tagged with #FOAMed, posted between January 1, 2020–December 31, 2024. Each month, the 100 tweets were selected from Twitter’s “Top” tab and manually curated. We used latent Dirichlet allocation (LDA) to identify thematic clusters. Hashtag usage and engagement metrics were assessed using descriptive statistics and linear regression.

**Results:**

We identified 10 distinct topics were identified through LDA modeling: point-of-care ultrasound (POCUS) education; neuro-radiology, cardiology-electrocardiogram (ECG); nephrology; and intensive care unit; ultrasound; prehospital/policy; webinars and learning; resuscitation scenarios; pediatric imaging; medical student education; and critical care and publications. Topic prevalence shifted over time: Early tweets focused on COVID-19 and critical care, while later years showed increasing attention to prehospital care, diagnostics, and POCUS. Mean tweet engagement peaked in 2023 (236.9 ± 914.6). Notably, hashtags such as #POCUS and #MedEd showed substantial increases in both usage and engagement, with #MedEd reaching a peak mean engagement of 287.7. In contrast, COVID-19 declined steadily, both in frequency (from 126 tweets in 2020 to just six in 2023) and in engagement (mean: 67.1 → 18.5). Spearman correlation analysis revealed that hashtag count had a weak but statistically significant correlation with engagement (ρ = 0.047, *P* < .001), suggesting that content quality, rather than volume, was the primary driver of visibility.

**Conclusion:**

FOAMed discourse on Twitter/X remains dynamic, responsive to clinical priorities and shaped by peer interaction. Natural language processing and topic modeling are valuable tools to uncover longitudinal trends in digital medical education, reinforcing Twitter/X’s role in informal, real-time learning communities.

## INTRODUCTION

Over the past decade, free open access medical education (FOAMed) has emerged as a global movement aimed at democratizing medical knowledge. By leveraging open-access digital platforms, FOAMed empowers clinicians to bypass traditional academic gatekeeping and engage directly in the exchange of clinical insights, case-based discussions, and practical resources. Among these platforms, Twitter—recently rebranded as X—has served as a real-time hub for FOAMed activity, connecting healthcare professionals across specialties and geographies through the unifying hashtag #FOAMed.^1^ Despite the scale and visibility of FOAMed discourse, there remains a notable lack of longitudinal, data-driven studies examining how its content and engagement dynamics have evolved over time. Most existing research has focused on narrow slices of activity, such as the COVID-19 pandemic, or on specific user groups, leaving broader patterns unexplored.^2^ Understanding the trajectory of FOAMed content is essential for identifying shifts in clinical emphasis and educational demand, thereby enabling educators and content developers to align resources with emerging areas of interest.^3^,^4^

To address this gap, we conducted a five-year analysis of 6,000 high engagement tweets tagged with #FOAMed, applying natural language processing (NLP) and topic modeling techniques. Our goal was to identify dominant themes, chart engagement trends, and map the evolution of hashtag usage, highlighting how online medical discourse has shifted during a transformative period in global healthcare. Given the expanding influence of social media on informal medical education, tracking shifts in thematic content over time may provide meaningful insight into evolving clinical and educational priorities.

## METHODS

### Study Design

In this retrospective, descriptive bibliometric study we aimed to analyze the thematic and engagement characteristics of tweets tagged with #FOAMed between January 1, 2020–December 31, 2024. A total of 100 tweets per month were manually curated from the ‘Top’ tab in Twitter’s search results, resulting in a dataset of 6,000 tweets across the five-year study period. This sampling strategy was selected to ensure consistent temporal coverage and to reflect content highlighted by Twitter’s own ranking algorithm, in the absence of a publicly accessible full archive of FOAMed tweets.

### Data Collection Process

We retrieved tweets using Twitter’s native search interface with the following structured query format: https://twitter.com/search?q=%23FOAMed%20since:YYYY-MM-DD%20until:YYYY-MM-DD&src=typed_query&f=top. The first 100 English-language tweets appearing in the “Top” tab each month were manually selected using Twitter’s public search interface. Although the specific criteria behind the “Top” tab rankings are not publicly disclosed, they are believed to incorporate signals such as recency, user relevance, and visible engagement metrics. We entered each selected tweet was into a structured Excel spreadsheet (Microsoft Corporation, Redmond, WA) and reviewed them for eligibility. Although account-level data (e.g, usernames, follower counts) were available, we excluded them from analysis to avoid ethical concerns and to maintain our focus on content-level engagement.

**Population Health Research Capsule**

What do we already know about this issue?

*Free Open Access Medical Education (FOAMed) provides online medical education, but long-term trends in engagement and thematic focus on Twitter/X are not well characterized*.

What was the research question?

*How have FOAMed tweet themes and engagement patterns evolved over five years (2020–2024)?*

What was the major finding of the study?

*Median engagement rose 34→57 (2020–23) and then declined to 42 (2024); #POCUS increased; #COVID19 declined*.

How does this improve population health?

*Tracking FOAMed trends highlights shifting educational priorities, enabling alignment of open-access resources with evolving clinical needs*.

### Inclusion Criteria

We included tweets if they 1) contained the hashtag #FOAMed, 2) were written in English, 3) were posted between January 1, 2020–December 31, 2024, 4) displayed visible engagement metrics (likes, retweets, replies), and (5) were not duplicated within the monthly datasets. Tweets that failed to meet any of these criteria were excluded. All tweets inherently met criterion (4) since the “Top” tab only displays posts with visible engagement.

### Variables and Dataset Structure

For each tweet, we extracted the following variables: username; date of posting; tweet content; engagement metrics (number of likes, retweets, and replies); all hashtags used; and the total number of hashtags. All monthly datasets were merged into a single master dataset comprising 6,000 unique tweets, each represented as a structured row with corresponding variables. We did not apply bot-detection algorithms, and engagement metrics were recorded as displayed by Twitter, without adjustments.

### Text Preprocessing and Topic Modeling

Natural language processing techniques were implemented in Python v3.11 (Python Software Foundation, Wilmington, DE). Preprocessing included lowercasing, removal of punctuation and uniform resource locators (URL), and stopword elimination using an extended list based on the Natural Language Toolkit (NLTK) corpus, augmented with FOAM-specific terms (eg, foamed, foamcc, foamrad). We used the resulting cleaned and tokenized corpus as input for topic modeling.

We conducted topic modeling using latent Dirichlet allocation (LDA) implemented via the Gensim library. Models with 6–10 topics were tested using a bag-of-words representation. We selected the optimal model based on the highest c_v coherence score (0.4507), which yielded 10 topics. Each tweet was assigned a dominant topic based on its highest posterior probability. Human-readable topic labels were manually assigned by interpreting the top 10 keywords for each topic. We then used these topic assignments for temporal and statistical analysis.

### Hashtag Analysis and Engagement Trends

Hashtags were extracted using regular expressions. We analyzed their frequency and distribution on a monthly and annual basis. Hashtag usage was also compared against engagement levels, defined as the sum of likes, retweets, and replies. We identified the top-performing tweets each year (based on likes, retweets, and replies), and their content was examined to explore potential drivers of high engagement.

### Statistical Analysis

We calculated descriptive statistics, including mean, median, standard deviation, and interquartile range (IQR), for engagement metrics and hashtag counts. Relationships between content features (eg, hashtag count, tweet length) and engagement were evaluated using Spearman correlation coefficients. We applied non-parametric tests (Mann-Whitney *U* and Kruskal-Wallis) for group comparisons, given the non-normal distribution of engagement metrics—driven by a small number of highly viral tweets—as confirmed by the Shapiro–Wilk test (*P* < .001).

To assess trends over time, we calculated the number of tweets assigned to each LDA topic annually. Linear regression was used with year as the independent variable and topic frequency as the dependent variable. We reported slope (indicating average annual change) and *R*^2^ values (goodness of fit) to determine whether each topic was increasing, decreasing, or stable over the study period. All analyses were conducted in Python v3.11 using the pandas, NumPy, SciPy, Seaborn, Scikit-learn, and Gensim libraries.

### Ethical Considerations

All data used in this study were publicly available and collected in compliance with Twitter’s terms of service. No identifiable private information was accessed, no human subjects were involved, and the analysis did not require interaction or intervention. Therefore, this study qualified was exempt from institutional board review under current guidelines.

## RESULTS

### Dataset Overview

We analyzed 6,000 English-language tweets tagged with #FOAMed, evenly distributed over five years (2020–2024) with 1,200 tweets sampled per year. After preprocessing, the median number of hashtags per tweet was four, while the median total engagement (likes + retweets + replies) was 42. However, the mean engagement was notably higher at 129.7 (SD 417.5), highlighting the impact of a small number of highly viral tweets on the overall distribution. The average number of hashtags per tweet was 5.18 (SD 4.07), with a maximum of 24. Interquartile range analysis revealed that 233 tweets exceeded the outlier threshold of 14.5 hashtags, contributing to a right-skewed distribution. Across the dataset, the median number of likes was 27 (Q1–Q3: 13–70), retweets had a median of 10 (Q1–Q3: 5–21), and replies had a median of 3 (Q1–Q3: 1–8). All annual comparisons showed statistically significant differences (*P* < .001) based on Kruskal-Wallis tests, as detailed in Table 1.

### Correlation Between Tweet Features and Engagement

**Figure 1:**
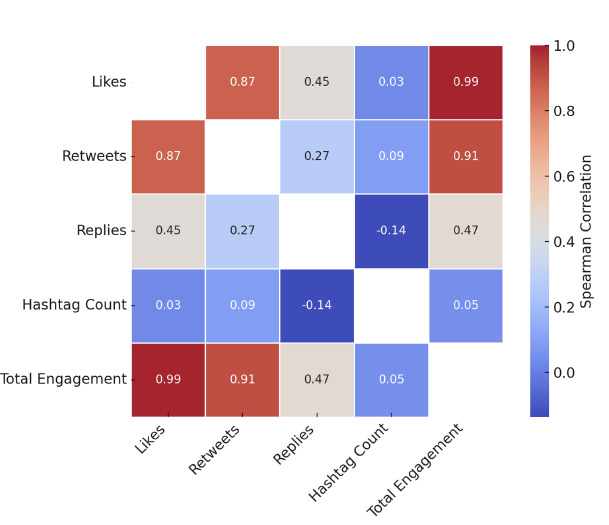
Distribution of likes, retweets, and replies across #FOAMed tweets (2020–2024) demonstrating the correlation patterns between individual engagement metrics and overall interaction levels in a five-year study of high engagement *FOAMed tweets. *FOAMed, free open access medical education.

Spearman correlation analysis revealed strong positive associations between likes (ρ = 0.991) and retweets (ρ = 0.911) with total engagement (P < .001 for both). The number of replies showed a moderate correlation (ρ = 0.465, P < .001). In contrast, hashtag count exhibited very weak correlations with total engagement (ρ = 0.047, P < .001) and with likes specifically (ρ = 0.033, P = .01) (Figure 1).

### Yearly Trends in Tweet Engagement

**Figure 2:**
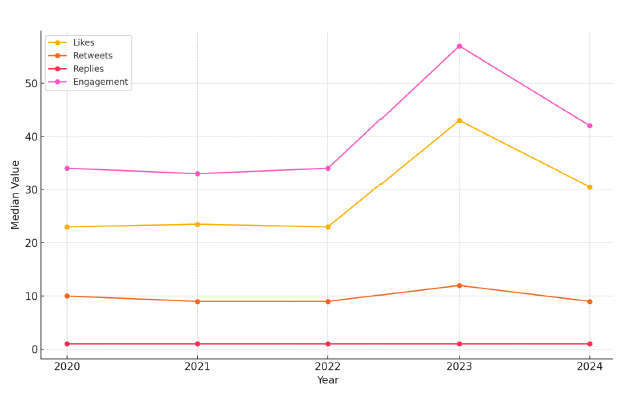
Yearly trends in mean and median engagement of #FOAMed tweets, illustrating the annual changes in average and median user engagement from 2020 to 2024, highlighting the disproportionate influence of highly viral tweets in 2023. *FOAMed*, free open access medical education.

Mean engagement per tweet steadily increased from 58.2 in 2020 to a peak of 236.9 in 2023, before declining to 149.6 in 2024. In contrast, median engagement values remained comparatively stable, ranging from 33.0 to 57.0 across the five-year period. This disparity suggests that a small subset of highly viral tweets disproportionately influenced the overall engagement trend (Figure 2).

### Most Common Hashtags (Excluding #FOAMed)

After removing the ubiquitous #FOAMed tag, analysis of the tweet corpus revealed several frequently used hashtags that reflect key thematic overlaps and clinical subdomains within the FOAMed community. The most prevalent was #meded, appearing in 2,009 tweets, followed by #medtwitter (1,667), #foamcc (1,295), #critcare (751), and #pocus (739). These hashtags covered a spectrum of topics, ranging from general medical education to specialty-specific content in critical care and point-of-care ultrasound. (POCUS). Line chart visualizations indicated a sharp increase in the use of #pocus and #critcare in 2023, while hashtags such as #covid19 and #foamcc peaked earlier, during 2020–2021.

**Figure 3:**
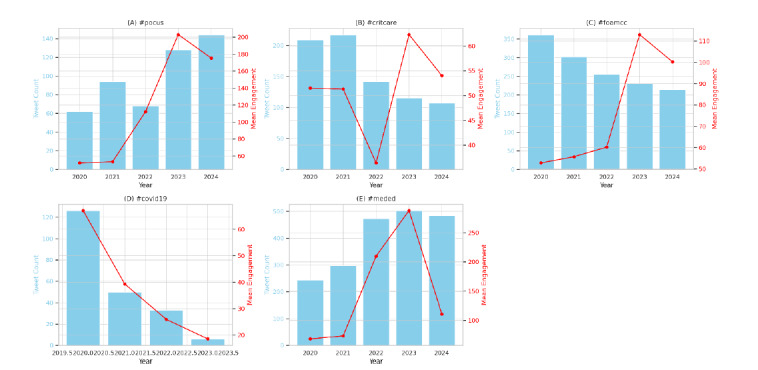
Yearly tweet frequency and mean engagement (composite) for five major hashtags. Notably, #pocus and #meded saw both usage and interaction peaks in 2023, while #covid19 steadily declined after 2020.

Hashtag-specific engagement metrics revealed differing interaction patterns. Tweets containing #pocus demonstrated a marked rise in both usage and engagement, with mean engagement increasing from 51.7 in 2020 to 203.0 in 2023, before a slight decline in 2024 (175.3). In contrast, #critcare remained stable in frequency but fluctuated around a moderate engagement range (36.4–62.3), suggesting plateaued user interest; #foamcc reached peak engagement in 2023 (mean: 113.0), paralleling a minor drop in tweet count. Notably, #covid19 declined both in frequency (from 126 tweets in 2020 to six in 2023) and engagement (mean: 67.1 to 18.5), reflecting waning attention to pandemic-related content. Conversely, #meded demonstrated consistent popularity and broad applicability, peaking in engagement at 287.7 in 2023 (Figure 3).

### Topic Modeling and Interpretation

Topic modeling yielded 10 thematically coherent categories, based on the dominant posterior probabilities assigned to each tweet. Manual review of the top keywords allowed for labeling of the following themes: POCUS education; neuroradiology, cardiology–electrocardiogram (ECG); nephrology and intensive care unit ultrasound; prehospital/policy; webinars and learning; resuscitation scenarios; pediatric imaging; medical student education; and critical care and publications. These topic labels were derived by interpreting the top 10 keywords for each category. A complete list is provided in Supplementary Table 1 to enhance transparency and interpretability.

### Yearly Distribution of Topics

**Figure 4:**
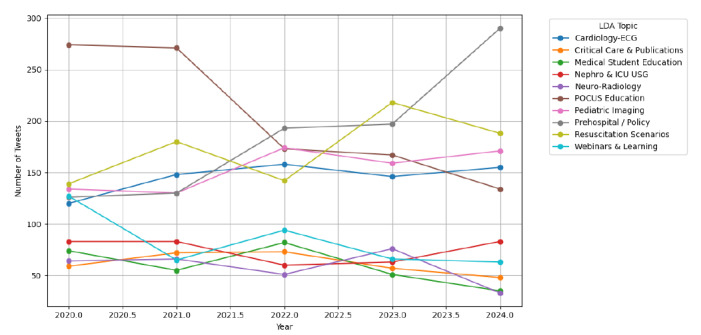
Topic distribution in FOAMed tweets over time. Latent Dirichlet allocation-derived topic frequencies from 2020–2024 reveal dynamic shifts in content focus. “Prehospital / policy” and “pediatric imaging” showed consistent upward trends, while early dominant themes like “POCUS education” declined. *ECG*, electrocardiogram; FOAMed, free open access medical education; *ICU*, intensive care unit; *LDA*, latent Dirichlet allocation; *USG*, ultrasound sonography; *POCUS*, point-of-care ultrasound.

Temporal visualization of topic prevalence (Figure 4) revealed substantial year-to-year variability, highlighting the dynamic and evolving nature of FOAMed discourse. The prehospital/policy category exhibited the most pronounced increase, rising from near-zero mentions in 2020 to over 190 tweets in 2024. Similarly, both resuscitation scenarios and pediatric imaging showed steady upward trends over the five-year period, reflecting growing engagement with emergent care protocols and pediatric diagnostics. Cardiology–ECG topics also increased gradually.

In contrast, the frequency of tweets related to POCUS education and webinars and learning peaked during 2020–2021 and then declined. This decline may reflect a shift back to in-person clinical education as the acute phase of the COVID-19 pandemic subsided.

### Statistical Trend Analysis

To explore how topic frequencies changed over time, we tracked yearly tweet counts for each category (Table 2). Four topics showed a clear upward trend over the five-year period, while one topic declined. Topics that showed little variation across years were described as stable.

## DISCUSSION

The FOAMed movement on Twitter/X represents a dynamic and widely engaged medical education community, with contributors spanning multiple specialties and regions. Our five-year analysis revealed content spanning several distinct clinical and educational themes, echoing prior observations that FOAMed tweets convey varied resources, expert advice, and personal insights.^1^ Through unsupervised topic modeling (LDA), we identified 10 distinct thematic domains, such as POCUS, cardiology, and critical care, underscoring the breadth of subjects circulating under the #FOAMed umbrella.

Temporal trend analysis highlighted shifts in topic prevalence over time. Discussions surrounding critical care and COVID-19 peaked during 2020–2021, subsequently tapering off, while topics such as ultrasound (#POCUS) surged in later years. This suggests a reorientation toward core clinical education as the pandemic receded. Such findings align with broader analyses of Twitter discourse during COVID-19, where content prevalence was closely tied to real-world events and often characterized by short-lived spikes.^5^ For instance, nearly 40% of FOAMed tweets in early 2020 focused on COVID-19, demonstrating the community’s rapid pivot to the crisis.^1^ As that urgency waned, conversations recalibrated toward longstanding topics such as resuscitation and diagnostics, illustrating the dynamic and event-responsive nature of this community-driven curriculum.

Hashtag use patterns further reinforce FOAMed’s adaptability. In tweets containing the #FOAMed tag, the most frequently co-occurring hashtags were #MedEd and #MedTwitter, indicating strong convergence between FOAMed and the broader medical education ecosystem. This overlap is unsurprising, given their shared ethos of open knowledge exchange. More specialized tags, such as #critcare and #POCUS, rose sharply in 2023, reflecting increased focus on critical care and ultrasound training. Conversely, #COVID19 and offshoot hashtags like #FOAMcc peaked early in the study period, consistent with an initial pandemic response followed by a return to non-COVID-19 content.

Engagement with FOAMed tweets remained robust throughout 2020–2024, with a notable uptick in average interactions by 2023. This may reflect a reinforcing pattern observed in other social media–based educational settings, where frequently shared or interacted content tends to receive additional visibility, regardless of initial source or status.^6^ Previous research has shown that FOAMed thrives on interactivity. Riddell et al reported that 80% of tweets by influential emergency physicians elicited replies, retweets, or likes, underscoring strong community engagement.^7^ In our analysis, a small number of tweets with relatively high engagement appeared to drive the annual mean upward—typically those featuring novel clinical insights, concise infographics, or spirited debates. These characteristics align with prior findings that clarity and visual appeal significantly influence a tweet’s reach.^8^ In contrast, merely stacking hashtags or mentions was insufficient to guarantee engagement. Our data showed only a weak correlation between hashtag count and interaction, reinforcing that substance often outweighs superficial features.

The Twitter-based #FOAMed community is further sustained by a core network of influencers and content curators. Even without formal hierarchy, contributors worldwide collaborated to disseminate knowledge during the pandemic.^1^ Earlier work identified emergency medicine “hub” users—those with high in-degree or centrality metrics—who helped bridge subcommunities and extend content reach.^9^ We deliberately excluded user-level metadata (eg, follower count, author status) to maintain a strict focus on content-level engagement patterns. While we did not analyze user networks, prior research has shown that influential voices can help amplify educational content in online communities. Still, FOAMed is not a one-way broadcast by experts; it cultivates bidirectional discourse. A mixed-methods analysis of EM tweets found that physicians frequently replied, questioned, and even engaged in informal banter, thereby “building social rapport” within a professional context.^7^ This aligns with Wenger’s framework of a “community of practice” and may contribute to the sustained vitality of the platform. In related studies, user participation, whether active or passive, was linked to perceived value and satisfaction.^10^ Even “lurkers” gained from the communal pool of knowledge and ongoing dialogue.

Importantly, our study illustrates how modern data science can enhance the understanding of digital medical education. By leveraging NLP and topic modeling, we systematically analyzed thousands of tweets to extract dominant themes and patterns. This builds on earlier work that relied on manual coding or sentiment analysis to study Twitter-based FOAMed content.^1^,^2^ At scale, automated topic modeling methods such as LDA have been shown to detect latent, clinically relevant themes in large text datasets that human reviewers might overlook, for instance enabling clinically interpretable topic extraction from referral letters or social work notes —demonstrating capabilities beyond what manual review typically achieves.^3^,^4^ The thematic categories we identified closely mirror known areas of educational focus within emergency and critical care medicine, reinforcing the validity of the method. Furthermore, tracking the trajectory of these themes offered a novel form of “digital epidemiology,” allowing us to observe shifts in learning priorities in near real time.

This approach resonates with prior findings from conference-based Twitter analyses. Yiu et al showed that tweet volume during a national emergency medicine conference did not correlate with formal speaker evaluations yet accurately reflected which sessions resonated with the audience.^11^ Similarly, our analysis suggests that Twitter/X can function as an informal but responsive barometer of educational relevance. In a post-pandemic landscape where medical journals and institutions increasingly recognize social media scholarship, from visual abstracts to tweetorials, FOAMed’s trajectory offers valuable insights for future digital learning strategies.

In summary, the FOAMed ecosystem remains dynamic and community-driven, with shifting content trends, engagement patterns, and evolving priorities. As new platforms and modalities emerge, the foundational principles of openness, collaboration, and responsiveness that underpin FOAMed should continue to inform innovations in medical education.

## LIMITATIONS

This study has several limitations that warrant consideration. First, the dataset was constructed from the “Top” tab of Twitter/X’s search results, which are ranked by a proprietary and non-transparent algorithm. This introduces potential selection bias, as high-engagement or influencer-driven content may be over-represented. Second, only English-language tweets were included, which limits the global representativeness of FOAMed content and may exclude valuable contributions from non-English-speaking communities. Third, although LDA topic modeling offers a scalable method for identifying thematic trends, it lacks the contextual nuance of qualitative analysis. Assigning human-readable labels to topics, while informed by keyword distributions, remains a subjective process.

Finally, the use of engagement metrics (likes, retweets, replies) as indicators of influence should be interpreted cautiously. These metrics reflect attention and visibility, but not necessarily educational impact, knowledge retention, or learning outcomes. We also observed a relative drop in engagement in 2024 compared to 2023, which may reflect broader platform trends, such as the rebranding of Twitter to X or reduced user activity. However, our study was not designed to evaluate such factors.

## CONCLUSION

This five-year bibliometric- and natural language processing-based analysis provides a detailed view of how #FOAMed content on Twitter/X has evolved in response to clinical trends, global events, and shifting user interests. Our findings reveal that the Twitter-based #FOAMed community is thematically diverse, highly responsive, and sustained by a core network of engaged contributors. As content transitioned from pandemic-related topics to core clinical themes, we observed clear patterns in both topic distribution and engagement dynamics. By applying modern analytical tools, particularly topic modeling, this study contributes both methodologically and conceptually to the field of digital medical education scholarship. The results suggest that platforms like Twitter/X function not only as channels for information dissemination but also as real-time indicators of professional priorities and peer-driven learning.

Looking forward, future research should explore regional variations in FOAMed discourse, compare activity across platforms (eg, Mastodon, Bluesky), and integrate qualitative methods such as sentiment analysis or network mapping. Importantly, distinguishing educational value from mere virality will be essential in understanding how informal learning truly occurs in these digital spaces. Ultimately, FOAMed’s ethos of open access, collaboration, and agility positions it to remain a vital force in the evolving landscape of medical education.

## Supplementary Information



## Figures and Tables

**Table 1:** Descriptive statistics for tweet engagement and content features across 6,000 #FOAMed tweets between 2020–2024.

Tweet Metrics (n)	2020 (1,200)	2021 (1,200)	2022 (1,200)	2023 (1,200)	2024 (1,200)	P-value
Likes (mean ± SD)	39.9 ± 76.5	53.1 ± 160.7	101.6 ± 269.4	185.7 ± 750.9	117.2 ± 301.0	N/A
Likes (median [Q1–Q3])	23 (13 – 43)	23 (13 – 51)	23 (11 – 77)	43 (19 – 124)	30 (15 – 89)	< .001
Retweets (mean ± SD)	16.3 ± 36.1	16.5 ± 36.4	27.8 ± 64.7	45.7 ± 151.6	29.0 ± 62.5	N/A
Retweets (median [Q1–Q3])	10 (5 – 19)	9 (5 – 18)	9 (4 – 21)	12 (5 – 31)	9 (4 – 24)	< .001
Replies (mean ± SD)	2.04 ± 4.47	2.23 ± 5.92	2.7 ± 7.45	5.51 ± 19.34	3.42 ± 14.12	N/A
Replies (median [Q1–Q3])	1 (0 – 2)	1 (0 – 2)	1 (0 – 2)	1 (0 – 4)	1 (0 – 2)	< .001
Engagement (mean ± SD)	58.2 ± 112.2	71.8 ± 200.8	132.1 ± 337.4	236.9 ± 914.6	149.6 ± 371.7	N/A
Engagement (median [Q1–Q3])	34 (20 – 64)	33 (18 – 72)	34 (17 – 104)	57 (26 – 161)	42 (20 – 117)	< .001
Hashtag Count (mean ± SD)	5.32 ± 4.28	5.32 ± 4.17	5.7 ± 4.12	4.95 ± 3.31	5.8 ± 4.28	N/A
Hashtag Count (median [Q1–Q3])	4 (2 – 7)	4 (2 – 8)	4 (3 – 8)	4 (3 – 6)	4 (3 – 8)	< .001

Note: Kruskal-Wallis *P*-values are reported only for medians; mean ± SD rows are marked as N/A.(non-parametric, Shapiro-Wilk test < .001)

*FOAMed*, free open access medical education*; N/A*, not applicable.

**Table 2:** Linear trend analysis of topic frequencies (2020–2024)

Topic	Slope (tweets/year)	R^2^	Trend
Prehospital / policy	+39.5	0.881	Increasing
Resuscitation scenarios	+13.6	0.419	Increasing
Pediatric imaging	+10.3	0.628	Increasing
Cardiology-ECG	+6.8	0.512	Increasing
Nephro and ICU USG	−2.0	0.072	Decreasing

Linear regression analysis of yearly topic frequencies identified five themes with meaningful trends. “Prehospital / Policy” showed the strongest increase (*R*^2^ = 0.881), while «Nephro and ICU USG” slightly declined.

*ECG*, electrocardiogram; *ICU*, intensive care unit; *Nephro*, nephrology; *USG*, ultrasound sonography.
