# Supplementary material for: Mapping Five Years of #FOAMed: Trends, Engagement, and Shifting Topics on Twitter/X
Source: West J Emerg Med. 2025 Dec 19;27(1):25–32. doi: 10.5811/westjem.47392 (PMC12815566; doi:10.5811/westjem.47392)
Supplement: Supplementary file 1 [file wjem-27-25-s001.docx]

| **Topic Label** | **Top 10 Keywords** |
| --- | --- |
| **POCUS Education** | pocus, meded, medtwitter, answer, radres, new, give, echofirst, work, spoilers |
| **Neuro-Radiology** | anatomy, radres, mass, neurorad, radiology, radtwitter, neurology, imaging, meded, mri |
| **Cardiology–ECG** | meded, medtwitter, cardiotwitter, ecg, cardiology, cardioed, medx, medicine, usmle, medical |
| **Nephro & ICU USG** | pocus, pressure, nephrology, via, nephpearls, meded, ultrasound, intensive, find, palpitations |
| **Prehospital / Policy** | meded, medtwitter, gitwitter, surgery, paramedic, poll, eds, radiology, neisvoid, doctors |
| **Webinars & Learning** | pocus, free, meded, still, webinar, get, check, medtwitter, learn, new |
| **Resuscitation Scenarios** | answer, diagnosis, case, pain, podcast, medtwitter, meded, arrest, history, resus |
| **Pediatric Imaging** | shows, radres, pedsrad, left, raded, radiology, right, meded, abdominal, upper |
| **Medical Student Education** | pocus, medicalstudent, ebm, ping, podcast, shift, resident, emergencymedicine, whiteboardteaching, meded |
| **Critical Care & Publications** | critcare, openaccess, isicem, jlvincen, icu, bmc, read, article, patients, full |

**Supplementary Table 1 – LDA-Derived Topics and Top Keywords**
Ten topics were derived via Latent Dirichlet Allocation (LDA), each labeled based on the most frequent associated terms. Topics reflect dominant clinical and educational themes in #FOAMed discourse.
